# Supplementary material for: More emergency patients presenting with chest pain
Source: PLoS One. 2023 Mar 23;18(3):e0283454. doi: 10.1371/journal.pone.0283454 (PMC10035919; doi:10.1371/journal.pone.0283454)
Supplement: S1 Table — Specific diagnoses among patients to whom an ambulance was sent as urgency level A due to chest pain, and who subsequently were diagnosed with AMI. (DOCX) [file pone.0283454.s001.docx]

| **ICD-diagnoses** | **N (%)** |
| --- | --- |
| I21.4: Non-ST-elevation acute myocardial infarction | 807 (41.0) |
| I21.3: ST-elevation acute myocardial infarction | 553 (28.1) |
| I21.9: Acute myocardial infarction, unspecified | 362 (18.4) |
| I21.0: Anterior acute myocardial infarction with Q-wave development | 126 (6.4) |
| I21.1: Inferior or posterior acute myocardial infarction with Q-wave development | 121 (6.2) |
| **Total** | **1,969 (100)** |

Supplemental table S1: Diagnoses. Specific diagnoses among patients to whom an ambulance was sent as urgency level A due to chest pain, and who subsequently were diagnosed with AMI.
