# Supplementary material for: More emergency patients presenting with chest pain
Source: PLoS One. 2023 Mar 23;18(3):e0283454. doi: 10.1371/journal.pone.0283454 (PMC10035919; doi:10.1371/journal.pone.0283454)
Supplement: S2 Table — Mortality according to most frequent specific diagnoses among patients to whom an ambulance was sent as urgency level A due to chest pain. 48-hour mortality omitted due to low number of deaths (microdata). (DOCX) [file pone.0283454.s002.docx]

|  |  | **30-day mortality** | |
| --- | --- | --- | --- |
| **Diagnosis** | **Frequency, n** | **Number of deaths, n** | **Mortality, % (95% CI)** |
| **ICD-10 main chapter: Diseases of the circulatory system** | **6,430** | **232** | **3.6 (3.2-4.1)** |
| I20.9: Angina pectoris, unspecified | 982 | 5 | 0.5 (0.2-1.2) |
| I21.4: Non-ST-elevation acute myocardial infarction | 807 | 29 | 3.6 (2.5-5.1) |
| I48.9: Atrial fibrillation or flutter, unspecified | 588 | 7 | 1.2 (0.6-2.5) |
| I21.3: ST-elevation acute myocardial infarction | 553 | 19 | 3.4 (2.2-5.3) |
| I25.9: Chronic ischaemic heart disease, unspecified | 402 | 8 | 2.0 (1.0-3.9) |
| **ICD-10 main chapter: Respiratory diseases** | **1,072** | **78** | **7.3 (5.9-9)** |
| J18.9: Pneumonia, unspecified | 460 | 28 | 6.1 (4.2-8.7) |
| **ICD-10 main chapter: Symptoms and signs** | **5,288** | **41** | **0.8 (0.6-1.1)** |
| R07.4: Chest pain, unspecified | 3263 | 15 | 0.5 (0.3-0.8) |
| **ICD-10 main chapter: Other factors** | **3,634** | **37** | **1 (0.7-1.4)** |
| Z03.9: Observation for suspected disease or condition, unspecified | 2,014 | 29 | 1.4 (1.0-2.1) |
| Z03.5: Observation for other suspected cardiovascular diseases | 624 |  |  |
| Z03.4: Observation for suspected myocardial infarction | 430 |  |  |

Supplemental table S2: Mortality according to most frequent diagnosis. Mortality according to most frequent specific diagnoses among patients to whom an ambulance was sent as urgency level A due to chest pain. 48-hour mortality omitted due to low number of deaths (microdata)
