# Supplementary material for: More emergency patients presenting with chest pain
Source: PLoS One. 2023 Mar 23;18(3):e0283454. doi: 10.1371/journal.pone.0283454 (PMC10035919; doi:10.1371/journal.pone.0283454)
Supplement: S3 Table — Mortality according to the specific diagnoses with highest numbers of death among patients to whom an ambulance was sent as urgency level A due to chest pain. 48-hour mortality omitted due to low number of deaths (microdata). (DOCX) [file pone.0283454.s003.docx]

|  |  | **30-day mortality** | |
| --- | --- | --- | --- |
| **Diagnosis** | **Frequency, n** | **Number of deaths, n** | **Mortality, % (95% CI)** |
| **ICD-10 main chapter: Diseases of the circulatory system** | **6,430** | **232** | **3.6 (3.2-4.1)** |
| I46.9: Cardiac arrest, unspecified | 23 | 20 | 87.0 (70.3-96.7) |
| I21.3: ST-elevation acute myocardial infarction | 553 | 19 | 3.4 (2.2-5.3) |
| I21.4: Non-ST-elevation acute myocardial infarction | 807 | 29 | 3.6 (2.5-5.1) |
| I21.9: Acute myocardial infarction, unspecified | 362 | 25 | 6.9 (4.7-10.1) |
| I35.0: Aortic stenosis | 94 | 12 | 12.8 (7.5-21.4) |
| I71.0: Aortic aneurysm and dissection | 20 | 7 | 35.0 (18.5-59.7) |
| I21.0: Anterior acute myocardial infarction with Q-wave development | 54 | 6 | 11.1 (5.2-23.1) |
| **ICD-10 main chapter: Respiratory diseases** | **1,072** | **78** | **7.3 (5.9-9.0)** |
| J18.9: Pneumonia, unspecified | 460 | 28 | 6.1 (4.2-8.7) |
| J96.0: Acute respiratory failure | 36 | 16 | 44.4 (30.1-62.0) |
| J44.1: Chronic obstructive pulmonary disease with acute exacerbation, unspecified | 135 | 13 | 9.6 (5.7-16.0) |
| **ICD-10 main chapter: Symptoms and signs** | **5,288** | **41** | **0.8 (0.6-1.1)** |
| R07.4: Chest pain, unspecified | 3,263 | 15 | 0.5 (0.3-0.8) |
| **ICD-10 main chapter: Other factors** | **3,634** | **37** | **1.0 (0.7-1.4)** |
| Z03.9: Observation for suspected disease or condition, unspecified | 2,014 | 29 | 1.4 (1.0-2.1) |

Supplemental table S3: Mortality according to highest numbers of deaths. Mortality according to the specific diagnoses with highest numbers of death among patients to whom an ambulance was sent as urgency level A due to chest pain. 48-hour mortality omitted due to low number of deaths (microdata)
