# Supplementary material for: More emergency patients presenting with chest pain
Source: PLoS One. 2023 Mar 23;18(3):e0283454. doi: 10.1371/journal.pone.0283454 (PMC10035919; doi:10.1371/journal.pone.0283454)
Supplement: Attachment — Submitted filename: Review (1).docx [file pone.0283454.s004.docx]

In this retrospective population-based study, ‘More emergency patients presenting with chest pain’, the authors discuss an important topic. I commend them for their choice of topic especially its relevance to how we can use these findings as a scaffold to optimize healthcare services and make it more efficient. I do have a few comments for their consideration:

1. I urge the authors to proofread the manuscript again and eliminate the referencing errors in the text. There are some places where references are missing and need to be updated accordingly.
2. The relevance of the study is lacking in the text. Given that only a small proportion of patients with chest pain had an acute myocardial infarction and only a minority experienced fatal outcomes, it would be prudent to shed light on the fact of how the authors plan or at least suggest the application of these findings to optimize the healthcare delivery process.
3. The authors have not sub-classified the ICD-10 diagnosis of the patients with 48-hr and 30-day mortality. I would request them to add this to the text as it would lead to a more comprehensive understanding of the issues at hand.
4. The authors have done a great job at explaining the study methodology. However the result section at times is not coherent with the table the authors have given. It would be of great help if author made it more streamlined and thus make it tangible with the text.
