# Supplementary material for: More emergency patients presenting with chest pain
Source: PLoS One. 2023 Mar 23;18(3):e0283454. doi: 10.1371/journal.pone.0283454 (PMC10035919; doi:10.1371/journal.pone.0283454)
Supplement: Attachment — Submitted filename: Reviewers Comment.docx [file pone.0283454.s005.docx]

General Comments:

This original manuscript entitled, “More emergency patients presenting with chest pain”, discusses an essential and timely topic. I commend Lindskou et. al. for their choice of topic and study. I have a few comments for the authors to take into account.

Major Comments:

1. The choice of outcomes is interesting. Even though the outcomes including the number of ambulance dispatches for chest pain, patients diagnosed with AMI, and 48-hour and 30-day mortality in patients with chest pain are quite descriptive, I think the authors can perform a sub-group analysis comparing relevant outcomes like mortality, length of stay, complications, etc between patients presenting with chest pain vs non-chest pain patients.

2. Even though the topic of discussion here is well thought out and timely, I urge the authors to elaborate more on the significance and implications of this study.

3. In a similar study done by Pedersen et. Al. Done in Central Denmark the way of entrance into the EMS system was categorized as “112-requested” or “GP-requested”. (a) Under GP requested category, the prehospital response is based on the GPs’ clinical assessment rather than dispatch criteria. Excluding this patient strata may underestimate the prevalence of AMI in patients presenting with chest pain. Can the authors please elucidate why did they not consider this?

(a)	Pedersen CK, Stengaard C, Friesgaard K, Dodt KK, Søndergaard HM, Terkelsen CJ, et al. Chest pain in the ambulance; prevalence, causes, and outcome - a retrospective cohort study. Scandinavian Journal of Trauma, Resuscitation and Emergency Medicine. 2019;27(1):84.

4. Can the authors please be consistent in citing and referencing the manuscript? Also, on page 9 of the manuscript there is an error where reference sources cannot be found. Kindly address this.

5. Can the authors please address the grammatical errors and inconsistencies throughout the manuscript?
